# The Book World of Medicine and Science

**Published:** 1907-05-04

**Authors:** 


### THE BOOK WORLD OF MEDICINE AND SCIENCE.

A First Study of the Statistics of Pulmonary Tuberculosis. By Karl Pearson, F.R.S. (London: Dualau and Co. 3s. net.)

In this latest number of the Drapers' Company's Research Memoirs, Professor Pearson discusses the incidence of consumption as deduced from statistics furnished by a series of 300 life histories. He concludes that "the diathesis of pulmonary tuberculosis is certainly inherited . . . that for the artisan class the inheritance factor is far more important than the infection factor . . . that there is no reduced fertility in the case of tuberculous stocks, and, finally, that the elder offspring, especially the first and second, appear subject to tuberculosis at a very much higher rate than the younger members." These conclusions are opposed to the generally accepted teaching with regard to tuberculosis, but they are supported to the full by Professor Pearson's series of cases.

First Lines in Midwifery. By Ernest Herman, M.B., F.R.C.P. (London: Cassell and Company, Limited. New edition. 5s. net.)

The fact that this useful little guide to the essentials of obstetrics has reached its eleventh edition is a good index of its popularity with students. The new edition has been revised, and the author has added as an appendix the rules issued by the Central Midwives Board, with some useful explanatory notes and elucidations. It is in every way a practical, serviceable handbcok.

Eye Injuries and their Treatment. By A. Maitland Ramsay, M.D. (Glasgow: James Maclehose and Sons, 1907. Pp. x + 210, with numerous coloured plates and other illustrations. Price 18s. net.)

To review this book is a task both easy and pleasant. Whether considered as a contribution to technical knowledge, an exposition of sound practice, or an example of literary skill, it must be recognised as attaining a high standard of excellence. Still further, there are associated with the text several series of coloured plates possessing both clinical value and artistic merit. It is therefore evident that every effort has been made to make the book in all directions worthy of its theme. Possibly the title may suggest to some that the subjects discussed by Dr. Ramsay concern only a limited field of practice. But this is not the case. The slighter accidents which befall the eye and its appendages are not infrequent events in general practice, and the practitioner who can deal with them adequately, receives, and justly receives, considerable credit. Moreover, the more serious disasters demand that at least their nature and extent shall be recognised; and their ultimate results are often largely determined by an early diagnosis and by appropriate treatment. Hence every practitioner should be prepared to deal confidently and wisely with injuries to the eye, even though he may need in a certain number of cases the help of an ophthalmic surgeon. Dr. Ramsay's book is based upon a recognition of these truths. There is no event within the compass of his subject which is too trivial for discussion, and none so grave but that the record of his experience and advice may be welcomed. Hence the practitioner may be certain that with the help of this volume he will find the practical guidance necessary for the wise treatment of the various ophthalmic accidents, both great and small, which may occur in his practice. Great help in this direction will be obtained from the coloured plates, of the artistic merits of which we have already spoken. It only remains to say that next to attendance at an ophthalmic clinic these illustrations occupy the leading place. Their drawing, colouring, and teaching value, merit the highest praise. The whole book is planned with a practical aim, and the completeness with which details of treatment are described, and the care which is shown to make every statement both lucid and precise, are features worthy of cordial and and general appreciation. We have seldom reviewed a medical book which so faithfully and successfully achieves the purpose of its author.

